# Bilateral Cerebellar Hemorrhages: An Atypical Presentation of Acute Hemorrhagic Encephalomyelitis

**DOI:** 10.7759/cureus.64857

**Published:** 2024-07-18

**Authors:** Lovelina Singh, Shweta S Acharya, Praveen Arumugam, Suraj Shetti, Shalini Sharan

**Affiliations:** 1 Neurology, Max Super Speciality Hospital, New Delhi, IND; 2 Internal Medicine, Max Smart Super Speciality Hospital, New Delhi, IND; 3 Internal Medicine, Max Smart Super Speciality Hospital, Saket, New Delhi, IND; 4 Internal Medicine, Max Super Speciality Hospital, New Delhi, IND

**Keywords:** hurst disease, acute hemorrhagic leukoencephalitis (ahle), plasmapheresis treatment, seizure disorders, acute disseminated encephalomyelitis (adem)

## Abstract

Hurst disease, or Weston-Hurst syndrome, or acute hemorrhagic encephalomyelitis (AHEM), is an infrequent condition that usually gets provoked after a viral infection (respiratory tract infection) or, as reported in many case reports, post-vaccination. Hurst disease is a difficult-to-diagnose condition because it mimics common presentations such as stroke, decreased or loss of consciousness, brain abscess, and seizures, making clinical diagnosis challenging. Radiological imaging, such as magnetic resonance imaging (MRI) of the brain with diffusion-weighted imaging, now serves as the primary modality to identify such conditions, despite its lack of specificity. The treating doctor needs to do an in-depth analysis of the patient's history, as this carries a very high mortality rate. We hereby discuss a case that presented with seizures and deteriorating Glasgow Coma Scale (GCS) score, on imaging revealed posterior circulation acute hemorrhagic leukoencephalitis (AHLE)/AHEM, therein treated with steroids, plasmapheresis resulted in a good outcome implicating, early detection and timely management can reduce the mortality due to this condition.

## Introduction

Acute hemorrhagic encephalomyelitis (AHEM), otherwise called acute hemorrhagic leukoencephalitis (AHLE) or Weston-Hurst syndrome, is a rare form of demyelinating disease. Reports indicate a handful of cases with diverse forms of presentation. Though it is considered rare, the numbers have been booming since the COVID-19 vaccinations. The literature and case series by Ancau et al. [1] provide evidence that AHEM can manifest both post-viral infections and vaccine-induced complications. Although the exact mechanism is not completely understood, it may be explained by immune complex formation, vasculitis, cytokine release, endothelial activation, and molecular mimicry. We hereby present a rare disease that presents with unique symptoms such as seizures and neck pain, without any trigger factors mentioned previously. On further evaluation, she was diagnosed with AHEM. Therefore, neurologists and physicians must maintain a high level of diagnostic suspicion and promptly treat cases identified early in the disease's progression, given the value of time and the negative consequences of delayed diagnosis, which frequently result in sentinel events.

## Case presentation

**Figure 1:**
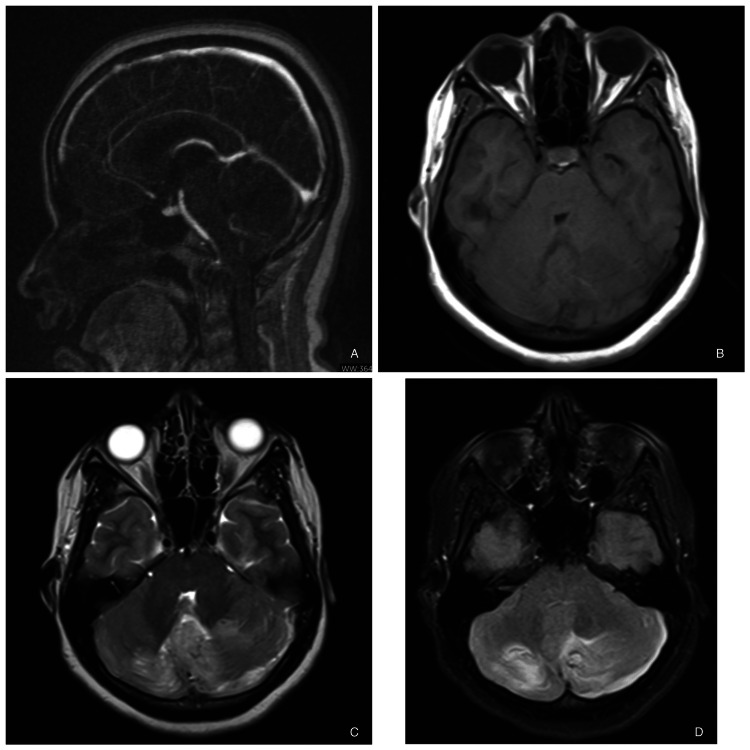
(A) MR venogram showing no signs of cerebral venous thrombosis, (B) MRI brain T1W image showing hyper-intensity in bilateral cerebellum, (C) MRI brain T2W image showing hyper-intensity in the same area, (D) MRI brain FLAIR showing hyperintensity in the same region as mentioned in T1. FLAIR - Fluid-attenuated inversion recovery

A female in her early 20s was brought in with an altered sensorium to the hospital emergency room. The attendants reported a sudden onset of neck pain, a headache that lasted for approximately 15 minutes, two episodes of vomiting, teeth clenching, eyeball rolling, and finally, unresponsiveness. There was no history of trauma, viral illness, or any recent vaccinations. She had no comorbidities or drug allergies. On examination, her airway was maintained, and her breathing was unlabored. She was hemodynamically stable with a Glasgow Coma Scale (GCS) of E3V1M5. Capillary blood glucose was within normal limits, and pupils were bilaterally constricted and sluggishly reacting to light. The respiratory system, cardiovascular system, and abdomen examination were unremarkable. On examination, the patient was in a post-ictal state; planters were flexor bilaterally, and power could not be assessed because of altered sensorium. Lorazepam sedated her for an MRI of the brain (Figures 1B-1D), which revealed confluent signal alteration in bilateral cerebellar hemispheres and vermis.

An MR venogram (Figure 1A) was performed to check for cerebral venous thrombosis but found no abnormality. She was admitted to the Neurosurgical Intensive Care Unit (NSICU). She had an episode of bradycardia; her pupils became mid-dilated, and her GCS dropped to E1V1M4. She was immediately intubated by the critical care medicine team and stabilized. An urgent computed tomography (CT) head was done to rule out acute hydrocephalus, which revealed bilateral cerebellar hemorrhages causing mass effect and effacement of the cerebellar folia and fourth ventricle, with mild upstream prominence of the bilateral lateral ventricles and third ventricle. Cerebral digital subtraction angiography (DSA) was done, which suggested no evidence of any intracranial AV malformation or aneurysm. Urgent suboccipital decompressive craniotomy was done with the partial evacuation of hematoma, and augmentation lax duraplasty was done under general anesthesia. Cerebellar tissue was very hard, and it became lax after the hematoma evacuation. Sampled tissue was sent for histopathology examination. She tolerated the surgery well, and post-operatively, she was shifted to the NSICU for post-op care. A post-surgery review of the non-contrast CT (NCCT) of the brain showed satisfactory decompression.

**Figure 2:**
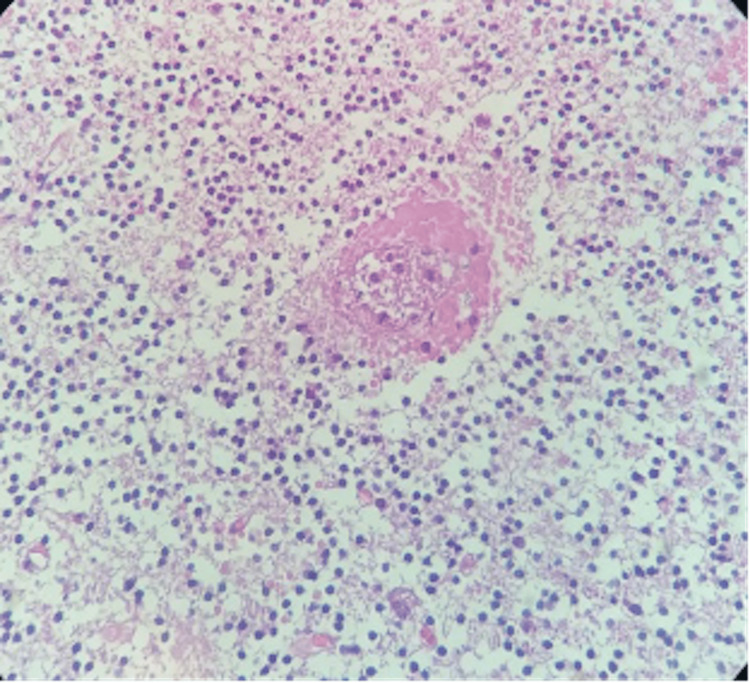
Histobiopsy of cerebellar parenchyma shows focal edema with spotty hemorrhages at places seen around small blood vessels. No inflammatory reaction, granuloma or necrosis is detected. IHC: CD45 - Negative for any significant leucocytic infiltrate in the cerebellar parenchyma. C4d – Negative on vessel walls.

Her initial routine laboratory investigations, such as blood counts and liver and kidney function tests, were within normal limits. Beta-HCG was negative. There were no laboratory reports suggestive of coagulopathy, and tests for viral serologies such as hepatitis C & B, HIV, dengue, and H1N1 were all negative. Cerebrospinal fluid (CSF) showed hemorrhagic fluid (hemorrhage extended to subarachnoid space). Her CSF reports showed normal total leucocyte cells, with predominant neutrophils; however, the protein was significantly raised to 422.8 mg/dL (normal range 15-40) with normal CSF glucose. The CSF culture was sterile and the fluid for the paraneoplastic panel, encephalitis panel, auto-immune panel, tuberculosis, and malignant cells were all negative. Meanwhile, her serum cold agglutinin was positive, but serum antinuclear antibody (ANA) and vasculitis LIA panel, ESR, C3, and C4 levels were all within normal limits. Histopathology examination of cerebellar tissue (Figure 2) revealed a hematoma and no abnormal findings.

The final diagnosis of AHEM was confirmed. She underwent three sessions of plasmapheresis and Intravenous methylprednisolone pulse therapy. However, the patient had persistent hydrocephalus, for which a ventriculoperitoneal (VP) shunt was placed. Later, she was tracheostomized because of the anticipated prolonged ventilation. Her sensorium improved gradually. She was shifted to a room later with a tracheostomy tube in situ on a portable ventilator. On follow-up, she was admitted for decannulation, observed with a portable non-invasive ventilation (NIV), and discharged with a hemodynamically stable condition. The patient's general well-being and ailments significantly improved over the course of five months with strict adherence to post-discharge care and constant rehabilitation.

## Discussion

Based on the results of the MRI, the differential diagnosis in our case consists of the following conditions: vasculitis, AV malformation, fulminant multiple sclerosis, acute disseminated encephalomyelitis (ADEM), AHLE, infectious encephalitis, and venous sinus thrombosis. A negative serologic and microbiological examination of the CSF combined with MRI findings of nonsuppurative, non-enhancing diffuse edematous lesions, primarily involving the cerebral hemispheres' white matter, could rule out infectious encephalitis, including herpes simplex encephalitis, and other viral, bacterial, or fungal infections.

Vasculitis presents with acute onset and emulates demyelinating disease and presents with tiny multiple lesions involving the cortices along with white matter. Various auto-immune profiles for vasculitis, but these findings were all negative in this patient. DSA was also done to rule out the same. Venous sinus thrombosis was excluded from MR venography. AV malformation was ruled out by DSA. Malignancy was ruled out as there were no malignant cells in the CSF, and the biopsy was normal. Distinguishing between fulminant multiple sclerosis, AHLE, and ADEM may be challenging. Nevertheless, there were no indications of demyelination in the biopsies.

The combination of the acute clinical course with CSF profiles, MRI findings, and lab findings strongly supports the diagnosis of acute hemorrhagic leukoencephalitis. Though the histobiopsy report was not consistent with the findings of AHLE, the patient did well after five courses of plasmapheresis. Her hospital stay was prolonged because of a tracheostomy and hospital-acquired infection, but her mRS was 0 after three months of presentation. This is the second case of its kind in the literature that survived after posterior circulation AHLE. In the literature, it was suggested to start plasmapheresis as soon as possible. To date, only one case of cerebellar AHLE has been reported to survive with an early start of plasmapheresis, as the exact mechanism is not clear, it is postulated that it removes humoral factors or circulating antibodies and alters the equilibrium of T helper type 1 and T-helper type 2 cells. Early diagnosis and strong suspicion of this rare disease are critical for starting treatment (plasmapheresis) and achieving a good functional outcome.

It is a rare and fulminant variant of ADEM. It was first described in 1941. AHLE is characterized by acute hemorrhagic lesions and prominent edema [2,3]. Since onset usually occurs after an infection, its pathophysiology is thought to involve either a molecular mimicry between infectious agent(s) and central nervous system (CNS) proteins or excessive inflammation triggered by a subclinical CNS infection or viral infection [2,3]. CSF is more inflammatory than ADEM, with polynuclear pleocytosis, blood cells, and a high rate of proteins [3,4].

In addition, the majority of such cases were observed in the pediatric population; AHEM, a rarer form of ADEM, had more mortality than the latter [5,6]. Pathological examination shows perivenous lymphocyte cuffing and fuzzy margin demyelinating lesions with microglial activation, typical of ADEM [3]. Furthermore, AHLE lesions have a prominent hemorrhagic focus, likely due to fibrinoid necrosis associated with polynuclear infiltration [3,7].

Most of the reported cases of posterior circulation AHLE were diagnosed on autopsy. The case reports published to date emphasize that this severe form of ADEM may present with a poor outcome and very high mortality, making this well-known condition a challenge. Due to its rare presentation, big studies or trials have not been published yet to guide the management of this rare and frequently fatal condition [8-10]. A boom in the incidence of this condition was observed and reported during the COVID-19 pandemic, especially post-vaccination. As per a systematic review and meta-synthesis by Manzo et al. [11], it was observed that there was an increase in the shift of median age to a more adult group with significantly increased hemorrhage on neuroimaging and mortality.

Management is usually treating the underlying pathology and several case reports, have reported favorable outcomes with different modes of immunosuppression, including high-dose corticosteroids, intravenous immunoglobulin, plasmapheresis, and cyclophosphamide [3,4,7-10,12]. The exact treatment guidelines have still not been established, due to its scarce presentation, possible lack of clear knowledge on pathogenesis, and a grim prognosis. Furthermore, it is important to acknowledge that more research and studies are required to understand and manage such unique conditions, wherein an early diagnosis and treatment can reduce mortality.

## Conclusions

Henceforth, early institution of treatment is strongly recommended on clinical suspicion rather than waiting for histopathological diagnosis. Aggressive therapeutic management is required to avoid fatal outcomes. Even though some authors have reported favorable neurologic outcomes in adult patients, the high rate of AHEM mortality necessitates a quick and aggressive treatment using combinations of decompressive surgery, corticosteroids, plasma exchange, and cyclophosphamide. Larger clinical studies are needed to further study the effect of TPE on neurologic outcomes in AHLE.
